# Pepsin enhances glycolysis to promote malignant transformation of vocal fold leukoplakia epithelial cells with dysplasia

**DOI:** 10.1007/s00405-022-07729-5

**Published:** 2022-11-16

**Authors:** Haitong Li, Shasha Zhang, Shuihong Zhou, Yangyang Bao, Xiaojuan Cao, Lifang Shen, Bin Xu, Weimin Gao, Yunzhen Luo

**Affiliations:** 1 Department of Otolaryngology, The Second Affiliated Hospital of Jiaxing University, Jiaxing, 314000 People’s Republic of China; 2 Department of Otolaryngology, College of Medicine, The First Affiliated Hospital, Zhejiang University, Hangzhou, 310003 People’s Republic of China

**Keywords:** Laryngeal cancer, Vocal fold leukoplakia, Malignant transformation, Pepsin, Glycolysis, GLUT1, HK-II

## Abstract

**Purpose:**

The mechanism underlying malignant transformation of vocal fold leukoplakia (VFL) and the precise role of the expression of pepsin in VFL remain unclear. This study aimed to investigate the effects of acidified pepsin on VFL epithelial cell growth and migration, and also identify pertinent molecular mechanisms.

**Methods:**

Immunochemistry and Western blotting were performed to measure glucose transporter type 1 (GLUT1), monocarboxylate transporters 4 (MCT4), and Hexokinase-II (HK-II) expressions. Cell viability, cell cycle, apoptosis, and migration were investigated by CCK-8 assay, flow cytometry and Transwell chamber assay, respectively. Glycolysis-related contents were determined using the corresponding kits. Mitochondrial HK-II was photographed under a confocal microscope using Mito-Tracker Red.

**Results:**

It was found: the expression of pepsin and proportion of pepsin+ cells in VFL increased with the increased dysplasia grade; acidified pepsin enhanced cell growth and migration capabilities of VFL epithelial cells, reduced mitochondrial respiratory chain complex I activity and oxidative phosphorylation, and enhanced aerobic glycolysis and GLUT1 expression in VFL epithelial cells; along with the transfection of GLUT1 overexpression plasmid, 18FFDG uptake, lactate secretion and growth and migration capabilities of VFL epithelial cell were increased; this effect was partially blocked by the glycolysis inhibitor 2-deoxy-glucose; acidified pepsin increased the expression of HK-II and enhanced its distribution in mitochondria of VFL epithelial cells.

**Conclusion:**

It was concluded that acidified pepsin enhances VFL epithelial cell growth and migration abilities by reducing mitochondrial respiratory complex I activity and promoting metabolic reprogramming from oxidative phosphorylation to aerobic glycolysis.

**Supplementary Information:**

The online version contains supplementary material available at 10.1007/s00405-022-07729-5.

## Introduction

Vocal fold leukoplakia (VFL) is a hyperplastic and keratosic stage of mucosal epithelial lesions in the vocal fold region, and its incidence rates considerably vary [1]. VFL has been suggested to be a precancerous lesion [2–4]. The risk of malignant transformation of the epithelium from VFL tissues significantly increases with the increase of dysplasia stage [5, 6]. However, the mechanism underlying the malignant transformation of VFL remains unclear.

Laryngopharyngeal reflux (LPR) evidently plays a key role in VFL development and malignant transformation [7, 8]. LPR contents include gastric acid as well as non-acid components. Pepsin (EC 3.4.23.1), a non-acid component, is a gastric proteolytic enzyme and is produced upon the activation of pepsinogen secreted from chief cells lining the gastric mucosa. Pepsin activity is the highest in acidic environments. Pepsin can reportedly enhance the epithelial–mesenchymal transformation of laryngeal cancer cells and also promote the migration and clonogenic ability of hypopharyngeal cancer cells, contributing to the progression of laryngeal and hypopharyngeal cancers, respectively [9, 10]. Ao et al.recently reported that long term administration of artificial pepsin-containing gastric juice to larynx induced hyperplasia of the laryngeal epithelium in normal mice [11]. Furthermore, the content of pepsin in VFL tissues has been found to be significantly higher than that in normal vocal fold tissues [12]. Another study reported that pepsin expression increases with dysplasia grade in patients with VFL [8]. However, the effects and mechanism of pepsin on the growth and migration abilities of VFL epithelial cells need to be further confirmed.

Glycolysis plays an important regulatory role in various malignant tumors, including laryngeal cancer. For instance, glycolysis is related to poor prognosis and radiotherapy resistance in patients with laryngeal cancer [13, 14]. Hexokinase-II (HK-II) inhibition can evidently improve radiosensitivity of laryngeal squamous cell carcinoma, which is related to the blocking of glycolysis [15, 16]. During malignant transformation, the demand of cells for nucleotides, lipids, amino acids, and energy increases significantly [17]. Accordingly, malignant transformed cells require more precursors, including glucose and glutamine. Glycolysis can supply intermediate metabolites for the de novo biosynthesis of amino acids, lipids, and nucleotides via the pentose phosphate pathway. During MYC proto-oncogene (Myc)- and harvey rat sarcoma virus oncogene (H-RAS)-mediated transformation of normal fibroblasts to malignant cells, the expression of multiple glycolysis-related regulatory enzymes, including HK-II and phosphofructokinase muscle (PFKM), gradually increase; consequently, glucose consumption and extracellular acidification rate (ECAR) also increase. The glycolysis inhibitor 2-deoxy-glucose (2-DG) has been found to markedly inhibit the cell activity of transformed cells [18]. GLUT1, a glucose transporter, plays a vital regulatory role in glycolysis. We previously found that high level of GLUT1 is associated with poor survival rate and radioresistance in laryngeal carcinoma [19–22]. It has also been demonstrated that GLUT1 participates in the development of precancerous lesions, including VFL [7, 23–25]. Thus, we speculate that pepsin and GLUT1 participate in VFL development by causing changes in glycolysis.

Herein, we aimed to investigate pepsin expression in VFL with different grades of dysplasia; furthermore, we evaluated the effects of acidified pepsin on the proliferation and migration capabilities of VFL epithelial cells. Our objective was to determine whether acidified pepsin enhanced the growth and migration capabilities of VFL epithelial cells by promoting metabolic reprogramming from oxidative phosphorylation to aerobic glycolysis.

## Materials and methods

### Clinical samples and ethics

Among patients who underwent surgery at The Second Affiliated Hospital of Jiaxing University, 5 adjacent tissues of VFL, 5 VFL without dysplasia (pure VFL), and 15 of VFL with mild (*n* = 5), moderate (*n* = 5), or severe (*n* = 5) dysplasia from different patients were assessed. This study was approved by the ethics committee of The Second Affiliated Hospital of Jiaxing University (ethics approval no.: jxey-20180015); written informed consent was obtained from each participant.

### Immunochemistry

5-μm slices were dewaxed in xylene solution, and hydrated in graded ethanol solutions. Antigen retrieval was conducted in an autoclave for 5 min followed by incubation with 3% hydrogen peroxide for 20 min. Immunohistochemistry were performed to detect the expression of pepsin (1:200, DF8591, Affinity Biosciences, Melbourne, AUS) in VFL tissues or adjacent tissues of VFL as described previously [26]. Subsequently, the slices were then observed under a microscope.

### Western blotting

Protein samples were extracted from cells or tissues with RIPA lysis buffer (Beyotime, P0013B, Shanghai, CN). Protein concentration was detected with a BCA Protein Assay Kit (Beyotime, P0012S, Shanghai, CN). Western blotting was prepared according to previous study[27] Primary antibodies against GAPDH (1:1000, AB-P-R 001, Hangzhou Xianzhi Biological Co., Ltd., Hangzhou, CN), pepsin (1:1000, DF8591, Affinity Biosciences, Melbourne, AUS), Bax (1:1000, 50599-2-Ig, San Ying Biotechnology, Wuhan, CN), Bcl-2 (1:1000, 26593-1-AP, San Ying Biotechnology, Wuhan, CN), caspase3 (1:1000, Ab184787, Abcam, Cambridge, UK), GLUT1 (1:1000, AF6731, Affinity Biosciences,Melbourne, AUS), MCT4 (1:1000, DF4182, Affinity Biosciences, Melbourne, AUS), and HK-II (1:1000, DF6176, Affinity Biosciences, Melbourne, AUS) were used to detect protein levels of these molecules. The next day, the membrane was soaked with secondary antibodies [HRP-labeled sheep anti-rabbit secondary antibody, 1:10000, BA1054, Wuhan Bode Bioengineering Co., Ltd., Wuhan, CN; goat anti-mouse IgG (H + L) HRP, 1:5000, S0002, Affinity Biosciences, Melbourne, AUS]. The membrane was subsequently scanned (Canon, K10486, Jap), and grayscale value of protein expression was analyzed by BandScan (Glyko, USA).

### Isolation of VFL epithelial cells

Briefly, fresh tissues obtained during surgery were quickly transferred to cold Dulbecco’s modified Eagle’s medium/F12 with penicillin–streptomycin, transported to the laboratory, and immediately treated. A digestion solution was prepared (0.25% trypsin plus 0.02% EDTA; 1:1) and warmed in an incubator. The tissue was excised into small pieces (0.5–1 mm^3^), immersed in this digestion solution, and incubated at 37 °C for 20 min. After centrifugation at 1000 rpm for 5 min, the supernatant was discarded, and the tissue was washed thrice with Hank’s solution. This process was repeated twice. The cells were suspended in a complete medium and passed through a 100-μm mesh filter. The remaining cells were suspended in Dulbecco’s modified Eagle’s medium with 1% penicillin–streptomycin and 10% fetal bovine serum. Subsequently, 5 × 10^5^ cells were inoculated in a 25-cm^2^ disposable culture flask, followed by incubation for 1 h at 37 °C in a humidified atmosphere of 5% CO_2_. The culture medium was changed every 2 days. The condition of the cells was observed under a phase contrast microscope every day.

### Cell transfection

Logarithmic growth phase cells (5 × 10^4^) were cultured at 37 °C and 5% CO_2_; Logarithmic growth phase cells (5 × 10^4^) were transfected with 2 μg plasmid using Lipofectamine™ 2000 (11668030, Invitrogen, US). After 6 h, the mixed solution was discarded and replaced with normal medium, followed by further culturing.

### CCK-8 assay

Cells (5 × 10^4^/mL) were added to a 96-well plate and cultured overnight at 37 °C in 5% CO_2_ incubator. Cells were transfected with vector (GenePharma, Shanghai, CN) or GLUT1 overexpression plasmid (GenePharma, Shanghai, CN). Subsequently, cells were treated with PBS (pH 7) plus pepsin (0.01, 0.1, and 0.5 mg/mL with pH 3 or 5, Sigma, cat no.: 1.07185, UK) or and 5 mM 2-DG [28] (D8375, Sigma, Shanghai, CN). After 72-h treatment, CCK-8 (HY-K0301, MCE, Shanghai, CN) was added (10 μL/well), followed by incubation. After 2 h, absorbance of each well was measured at 450 nm using a microplate reader.

### Cell cycle and apoptosis analysis

Cells were collected, washed twice with PBS, and centrifuged at 1000 rpm for 5 min. Cell cycle analysis was performed using the Cell Cycle kit (ab112116, Abcam, Cambridge, UK) and Apoptosis Analysis Kit (Yeasen, 40301ES50, Shanghai, CN). Cells were resuspended in 500 μL binding buffer; 10 μL propidium iodide (PI) (S6874, Selleck Chemicals, Houston, US) was then added in darkness at room temperature for 10 min. Cells were analyzed by flow cytometry.

### Detection of mitochondrial membrane potential

Mitochondrial membrane potential was estimated using JC-1 assay kit (C2006, Beyotime, Shanghai, CN) as described previously [29]. The JC-1 monomer and aggregate signals were detected by flow cytometry analysis.

### Cell migration

Cell migration capability was detected as per the method described by the previous study [30]. Finally, the upper chamber was fixed with 4% paraformaldehyde precooled on ice for 30 min, and stained with 0.1% crystal violet (C0121, Beyotime, Shanghai, CN) for 10 min. The images were photographed under a microscope.

### Detection of mitochondrial complex I activity

Mitochondrial complex I activity was detected using a kit (BC0515, Solarbio, Beijing, CN). 1 mL mitochondrial extract was added to 5 × 10^6^ cells, and the sample was homogenized on ice using a homogenizer or mortar, followed by centrifugation at 4 °C and 600*g* for 10 min. 400 μL extract buffer was added to the precipitate. The sample was then ultrasonically crushed (20% power, ultrasonic treatment for 5 s, interval of 10 s, repeated 15 times); the working solution was subsequently added, and optical density was measured at the wavelength of 340 nm to calculate mitochondrial complex I activity.

### Extracellular acidification rate (ECAR) and Oxygen consumption rate (OCR) detection

ECAR and OCR were measured in real-time with Glycolysis Stress Test Kit and Mito Stress Test Kit, respectively, using the Seahorse XFe96 Analyser (Seahorse Biosciences, USA) according to the previous study [31]. Data were normalized by cell numbers that was measured by the YO-PRO^®^-1 Assay (Thermo Fisher Scientific).

### Glucose uptake detection

The cells were cultured for 1 h and the glucose analog ^18^F-FDG (AM63503128, atomaxchem, ShenZhen, CN) was added. After culturing for 3 h, cell supernatant was rinsed with PBS, and this suspension was thoroughly mixed with the aforementioned supernatant. Trypsin was added to make the cells in each well float up, and digestion was terminated with culture media in time. The cells from each well were then collected and transferred into the tube. Intra- and extracellular radioactivity (Cin and Cout, respectively) were determined using a radioimmunoassay counter, and the ratio of Cin to Cout (Cin/Cout) was calculated.

### Lactic acid content detection

50 μL supernatant solution of VFL epithelial cells were collected. Subsequently, lactic acid content in VFL epithelial cells were measured using the lactic acid detection kit (ml1256935, Shanghai Meilian, Shanghai, CN) according to the manufacture’s instruction. The optical density of each well was measured at the wavelength of 450 nm.

### Cellular immunofluorescence assay

The cells were incubated with 50 nM Mito-Tracker Red (C1049, Beyotime, Shanghai, CN) for 30 min to label mitochondria. The primary antibody (HK-II, 1:200, DF6176, Affinity Biosciences, USA) was then added to incubate for overnight at 4 °C. Subsequently, the secondary antibody [goat anti-rabbit IgG (H + L) FITC conjugated, 1:200, S0008, Affinity Biosciences) was used for incubation at room temperature for 2 h. DAPI (C1002, Beyotime, Shanghai, CN) was used to stain nucleus in darkness for 5 min. After that, a sealing solution containing an anti-fluorescence-quenching agent (P0131, Beyotime, Shanghai, CN) was used to seal the slide. The co-localization of Mito-Tracker Red and HK-II were observed under a confocal microscope.

### Statistical analysis

Prism 9.0 was used for data analysis. Values represent mean ± SD. One-way analysis of variance (ANOVA) followed by least significant difference test was used for pairwise comparison between different groups. *P* < 0.05 was considered statistically significant.

## Results

### Pepsin content in VFL tissues increased with an increase in the grade of dysplasia

**Fig. 1:**
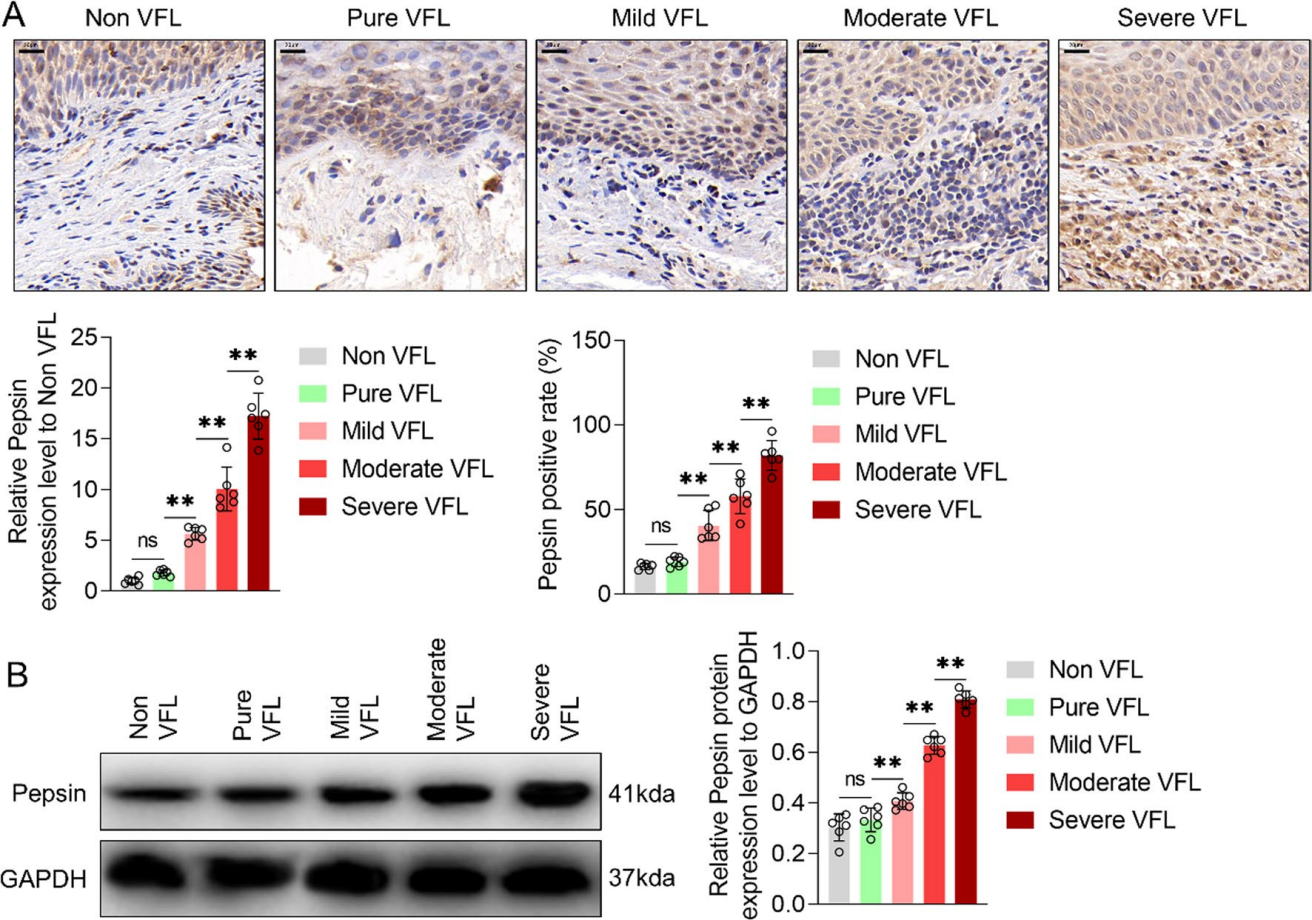
Pepsin protein content in vocal fold leukoplakia (VFL) with different grades of dysplasia. **A** Immunochemistry and **B** western blotting were performed to measure pepsin protein content. Proportion of pepsin^+^ cells was also detected. GAPDH was served as the internal reference. Bar graphs show strip gray values. *non-VFL* normal adjacent tissue of VFL, *pure VFL* VFL without dysplasia, *mild VFL* VFL with mild dysplasia, *moderate VFL* VFL with moderate dysplasia, *severe VFL* VFL with severe dysplasia. Scale, 20 μm. One-way ANOVA was used for statistical analysis. ns, *P* > 0.05; **P < 0.01. *n* = 6 cases/group

Immunochemistry and Western blotting were performed to determine differences in pepsin content in VFL tissues with different grades of dysplasia [adjacent tissues (i.e., distal non-VFL tissues, non VFL), VFL without dysplasia (pure VFL), and VFL with mild/moderate/severe dysplasia]. Immunochemistry results showed that VFL without dysplasia showed no significant changes (*P* > 0.05) in the content of pepsin or proportion of pepsin^+^ cells in comparison with distal non-VFL tissues. Furthermore, in comparison with VFL without dysplasia, VFL with mild/moderate/severe dysplasia showed significantly higher content of pepsin and greater proportion of pepsin^+^ cells (*P* < 0.01). In comparison with VFL with mild dysplasia, the content of pepsin and number of pepsin^+^ cells in VFL with moderate/severe dysplasia were significantly higher (*P* < 0.01). Besides, the content of pepsin and number of pepsin^+^ cells in VFL with severe dysplasia were higher than those in VFL with moderate dysplasia (*P* < 0.01) (Fig. 1A). Western blotting and immunochemistry findings were similar (Fig. 1B). Overall, these data indicated that pepsin content in VFL increased with the increase of the grade of dysplasia.

### Acidified pepsin enhanced the growth and migration of VFL epithelial cells

**Fig. 2:**
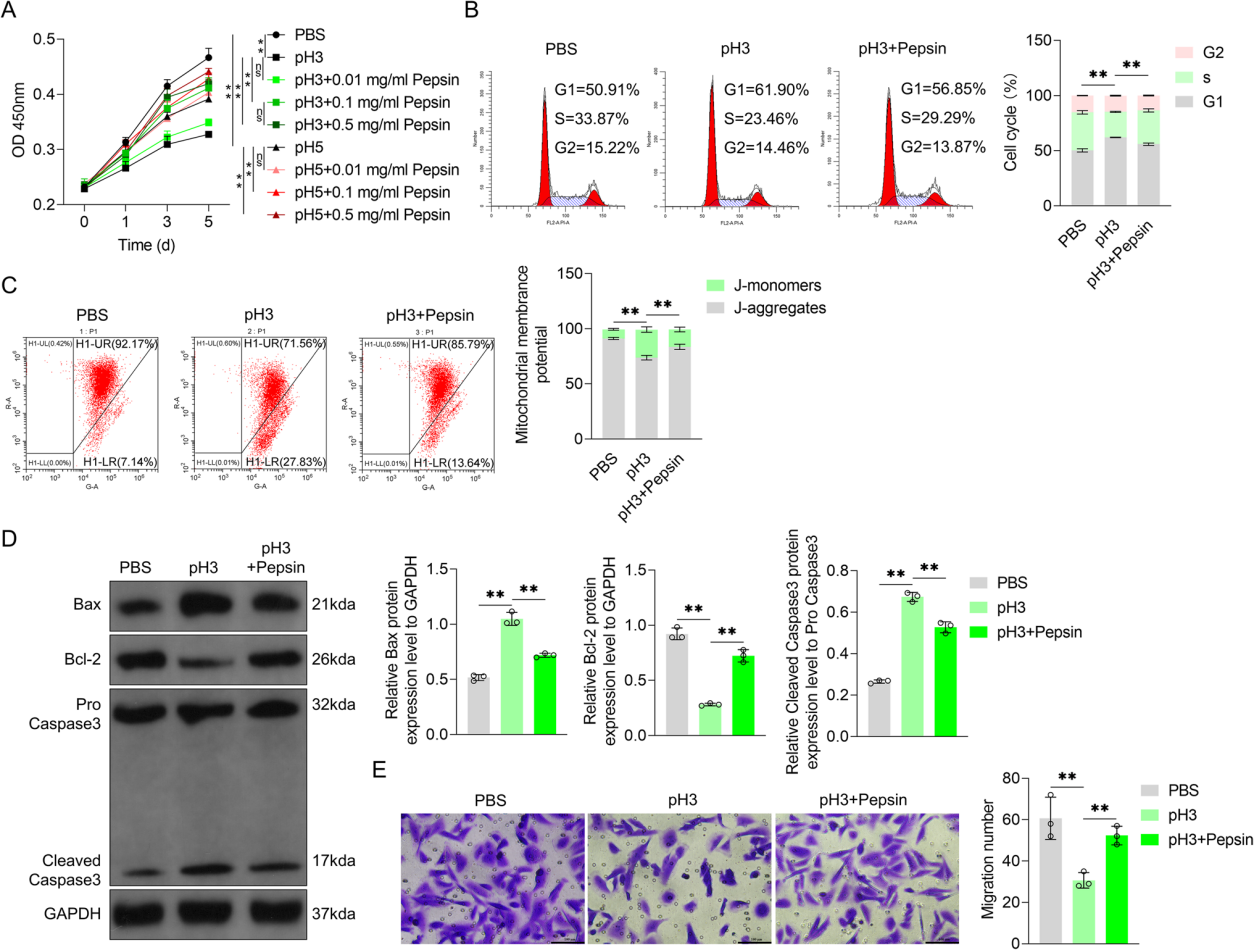
Effects of acidified pepsin on the growth and migration of VFL epithelial cells. **A** Effects of acidified medium (pH 3 or 5) and different acidified pepsin concentrations (pH 3 or 5; 0.01, 0.1, 1 mg/mL) on the activity of VFL epithelial cells were detected by CCK-8 assay. **B** PI staining combined with flow cytometry was used to detect the effects of acidified medium (pH 3) and acidified pepsin (pH 3, 0.1 mg/mL) on cell cycle distribution. **C** Effects of acidified medium (pH 3) or acidified pepsin (pH 3, 0.1 mg/mL) on mitochondrial membrane potential were detected by JC-1 probe and flow cytometry. **D** Effects of acidified medium (pH 3) and acidified pepsin (pH 3, 0.1 mg/mL) on caspase-3 shear and Bax and Bcl-2 expression were detected by Western blotting. GAPDH was served as the internal reference. On the right, strip gray values are shown. **E** Transwell chamber assay to detect the effects of acidified medium (pH 3) and acidified pepsin (pH 3, 0.1 mg/mL) on the migration ability of VFL epithelial cells. Scale, 100 μm. One-way ANOVA was used for statistical analysis. ns, *P* > 0.05; **P < 0.01

As pepsin presented the highest activity in acidic conditions, we treated VFL epithelial cells with acidified pepsin (pH 3 or 5) and detected changes in cell activity by performing CCK-8 assay to determine the role of pepsin in the proliferation of VFL epithelial cells. It was found that the activity of VFL epithelial cells in the treatment group (grown in acidified medium) showed a significant decrease, compared with that in the control group (grown in normal medium; group 1 vs. group 2; group 6 vs. group 1, *P* < 0.01). Furthermore, in comparison with the group treated with pH 3 media, different concentrations of acidified pepsin (pH 3, 0.1 mg/mL and above) significantly increased the activity of VFL epithelial cells (*P* < 0.01). The activity of these cells in 0.1 or 0.5 mg/mL acidified pepsin (pH 5) was significantly higher than that in pH 5 media (*P* < 0.01). The promoting effect on VFL epithelial cells at pH 3 and with 0.1 or 0.5 mg/mL pepsin was significantly higher than that at pH 5 and with 0.1 or 0.5 mg/mL pepsin (*P* < 0.01, Fig. 2A). In addition, there was no significant difference in the activity of VFL epithelial cells between 0.5 mg/mL and 0.1 mg/mL acidified pepsin (pH 3) (group 5 vs. group 4, *P* > 0.05, Fig. 2A). Thus, acidified pepsin (especially 0.1 and 0.5 mg/mL, pH 3) exerted a pro-proliferation effect on VFL epithelial cells compared to acidic conditions.

Pepsin and inactivated pepsin in normal medium were employed to confirm the role of acidified pepsin on the increased cell viability depending on its activity. The findings showed that both pepsin in normal medium and inactivated pepsin in normal medium could not affected cell viability of VFL epithelial cells (Fig. S1). Therefore, we thought that acidified pepsin-mediated the increase in growth and migration capabilities of VFL epithelial cells compared to acidic medium was potentially associated with the increased pepsin activity in acidic medium.

In addition to that, flow cytometry data revealed that in comparison with control medium, treatment with acidified medium increased the proportion of VFL epithelial cells in the G1 phase (*P* < 0.01, Fig. 2B). Furthermore, in comparison with acidified medium with pH 3, the proportion of the cells in the G1 phase after treatment with acidified pepsin (pH 3) was lower (*P* < 0.01, Fig. 2B). JC-1 assay data indicated that in comparison with control medium, treatment with acidified medium significantly increased the proportion of J-monomer and reduced the proportion of J-aggregates (*P* < 0.01, Fig. 2C). Besides, compared to treatment with acidified medium, the proportion of J-monomer in VFL epithelial cells decreased and that of J-aggregates increased upon treatment with acidified pepsin (*P* < 0.01, Fig. 2C). When mitochondrial membrane potential is high, JC-1 accumulates in the mitochondrial matrix and J-aggregates are formed; by contrast, when mitochondrial membrane potential is low, JC-1 exists as J-monomer as it cannot aggregate in the mitochondrial matrix. Therefore, acidified pepsin increased the mitochondrial membrane potential of VFL epithelial cells. Western blotting results showed that in comparison with control medium, caspase-3 cleavage and Bax expression significantly increased, while Bcl-2 content significantly decreased in acidified medium-treated VFL epithelial cells (*P* < 0.01, Fig. 2D). Compared to acidified medium, acidified pepsin significantly inhibited the cleavage of caspase-3 and the expression of Bax, and increased Bcl-2 content of VFL epithelial cells (*P* < 0.05, Fig. 2D). The migration ability of VFL epithelial cells in acidified medium was significantly lower than that in control medium (*P* < 0.01, Fig. 2E). Furthermore, the migration ability of the cells treated with acidified pepsin was significantly higher than that in acidified medium (*P* < 0.01, Fig. 2E). Overall, these data demonstrated that acidified pepsin significantly enhanced the growth and migration abilities of VFL epithelial cells.

### Acidified pepsin promoted the metabolic reprogramming of VFL epithelial cells from oxidative phosphorylation to glycolysis

**Fig. 3:**
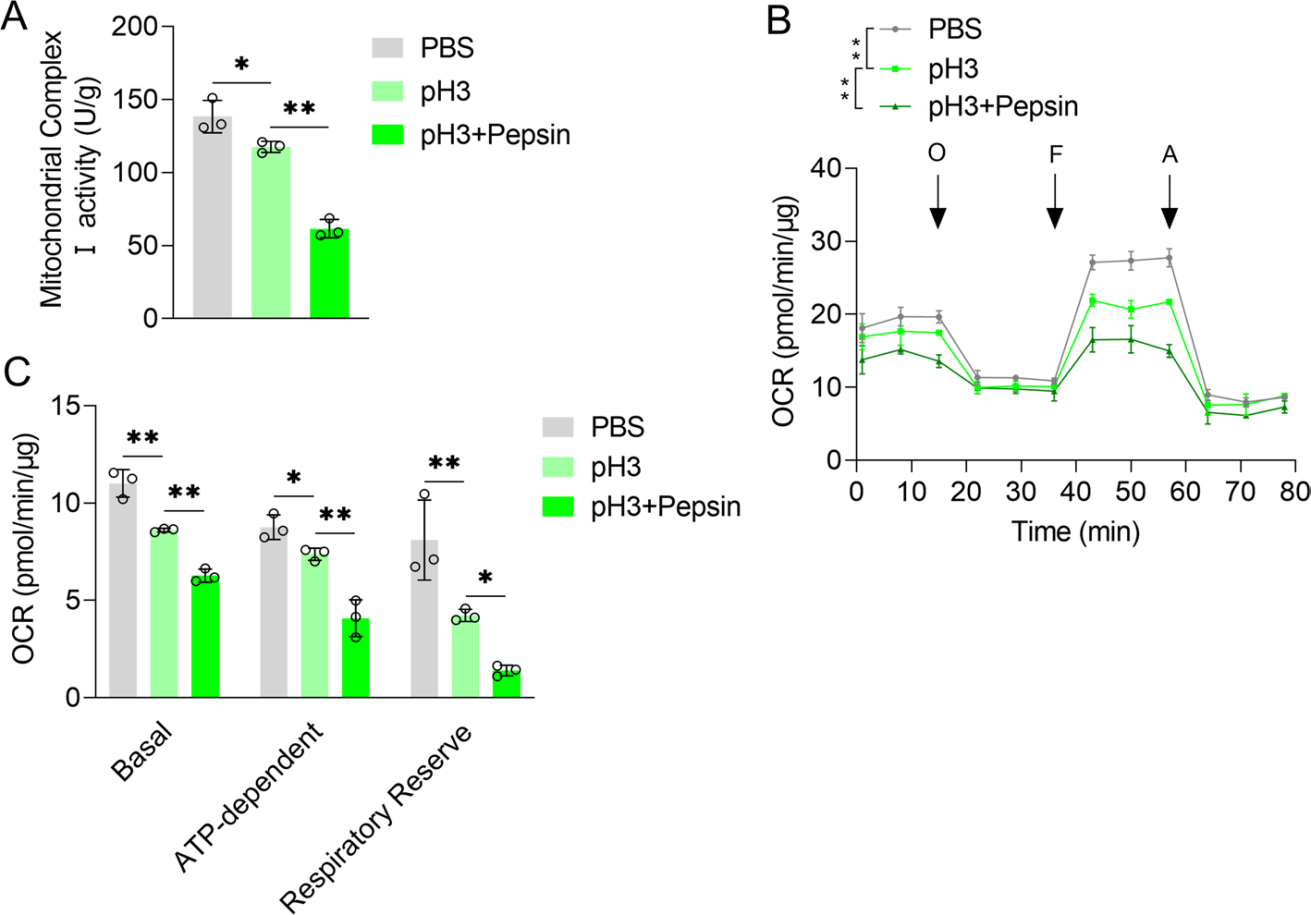
Effects of acidified pepsin on respiratory chain complex I activity and oxidative phosphorylation of VFL epithelial cells. **A** Effects of acidified medium (pH 3) and acidified pepsin (pH 3, 0.1 mg/mL) on mitochondrial respiratory chain complex I activity and **B** oxygen consumption rate (OCR). OCR of cells treated with ATP synthase inhibitor Oligomycin (O, 1 μM) or uncoupling agent carbonyl cyanide p-trifluoromethoxyphenylhydrazone (FCCP, F, 300 nM) or electron transport chain inhibitor rotenone/antimycin A (A, 1 μM) was detected. **C** Basic, ATP-dependent [difference between basic OCR and oligomycin-inhibited OCR], and Respiratory Reserve OCR [difference between FCCP-induced OCR and basal OCR] in B. One-way ANOVA was used for statistical analysis. *P < 0.05; **P < 0.01

In acidified medium, mitochondrial respiratory chain complex I activity of VFL epithelial cells was significantly lower than that in control medium (*P* < 0.01, Fig. 3A). Acidified pepsin significantly reduced this activity in comparison with acidified medium (*P* < 0.01, Fig. 3A). Oxygen consumption rate (OCR) results showed that the basic and ATP-dependent OCR, and respiratory reserve OCR values of VFL epithelial cells in acidified medium were lower than those in control medium (*P* < 0.01, Fig. 3B, C). Compared to control or acidified medium, acidified pepsin significantly reduced these OCR values (*P* < 0.01, Fig. 3B, C), indicating that acidified pepsin reduced oxidative phosphorylation of VFL epithelial cells.

**Fig. 4:**
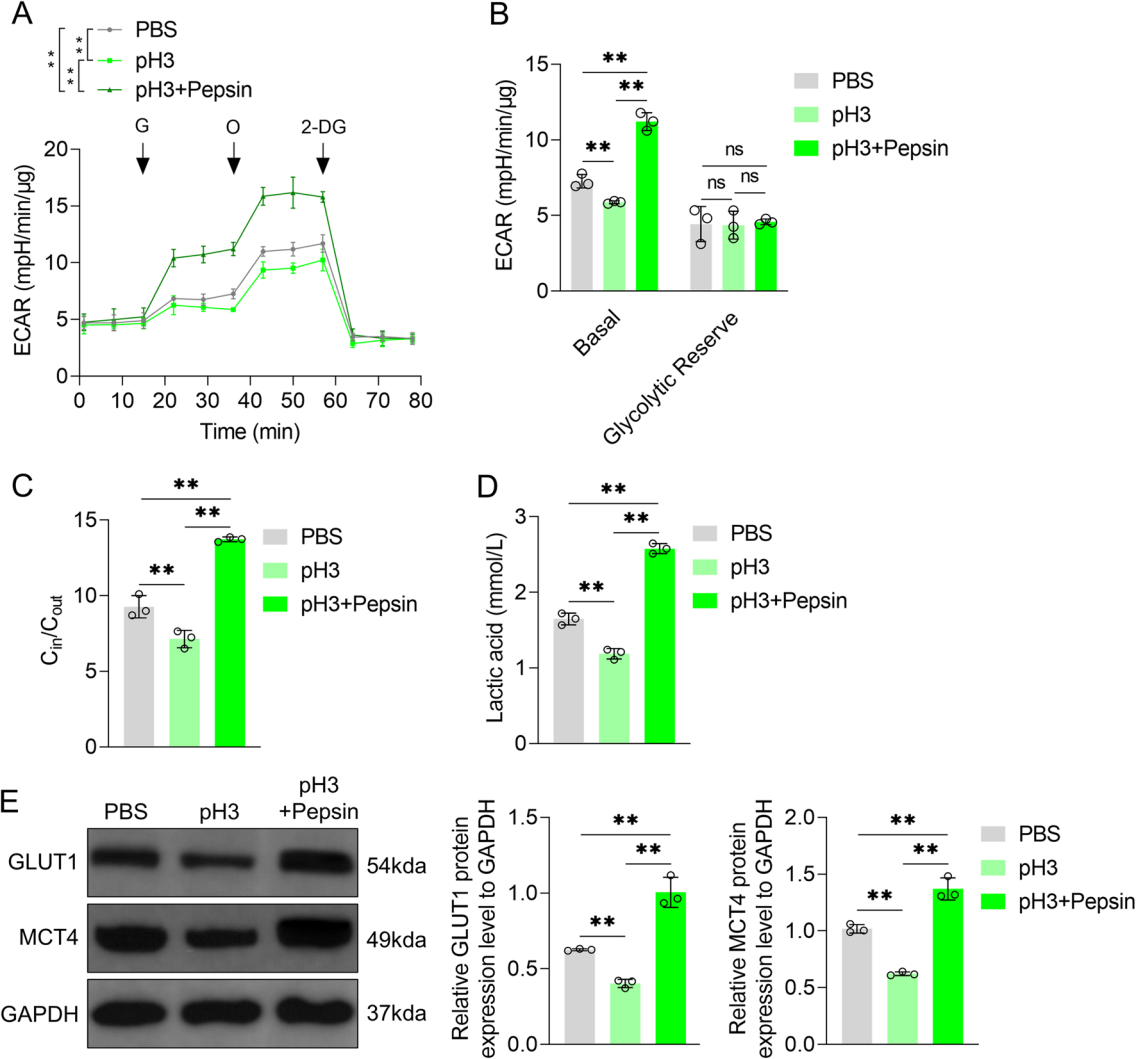
Effects of acidified pepsin on aerobic glycolysis of VFL epithelial cells. **A** Effects of acidified medium (pH 3) and acidified pepsin (pH 3, 0.1 mg/mL) on extracellular acidification rate (ECAR) of VFL epithelial cells treated with Glucose (G, 10 mM) or Oligomycin (O, 1 μM) or 2-DG (80 mM). **B** Basic ECAR and glycolytic reserve ECAR (difference between oligomycin-induced ECAR and basic ECAR) in **A**. **C** ^18^F-FDG was used to detect the effects of acidified medium (pH 3) and acidified pepsin (pH 3, 0.1 mg/mL) on glucose uptake. **D** Effects of acidified medium (pH 3) and acidified pepsin (pH 3, 0.1 mg/mL) on lactic acid and **E** glucose transporter type 1 (GLUT1), monocarboxylate transporters 4 (MCT4) protein contents, as detected by Western blotting. GAPDH was served as the internal reference. On the right, strip gray values are shown. One-way ANOVA was used for statistical analysis. **P < 0.01

Furthermore, compared to control medium, basic extracellular acidification rate (ECAR) value of VFL epithelial cells was significantly lower in acidified medium (*P* < 0.01, Fig. 4A, B); in contrast, glycolytic reserve ECAR value showed no significant differences (*P* > 0.05). Compared to control or acidified medium, acidified pepsin significantly increased basic ECAR value of VFL epithelial cells (*P* < 0.01, Fig. 4A, B). ^18^F-FDG uptake assay results showed that the glucose uptake ability of VFL epithelial cells in acidified medium was lower than that in control medium (*P* < 0.01, Fig. 4C). In addition, compared to control or acidified medium, acidified pepsin significantly increased this ability (*P* < 0.01, Fig. 4C). The findings of lactate secretion and ^18^F-FDG uptake assay were consistent (Fig. 4D). Western blotting data showed that glucose transporter type 1 (GLUT1) and monocarboxylate transporters 4 (MCT4) protein expression levels in VFL epithelial cells in acidified medium were lower than those in control medium (*P* < 0.01, Fig. 4E). In comparison with acidified medium, acidified pepsin increased GLUT1 and MCT-4 protein levels in VFL epithelial cells (*P* < 0.01, Fig. 4E). These results suggested that acidified pepsin promoted the metabolic reprogramming of VFL epithelial cells from oxidative phosphorylation to aerobic glycolysis by reducing mitochondrial respiratory chain complex I activity.

### GLUT1 enhanced glycolysis to promote the growth and migration of VFL epithelial cells

**Fig. 5:**
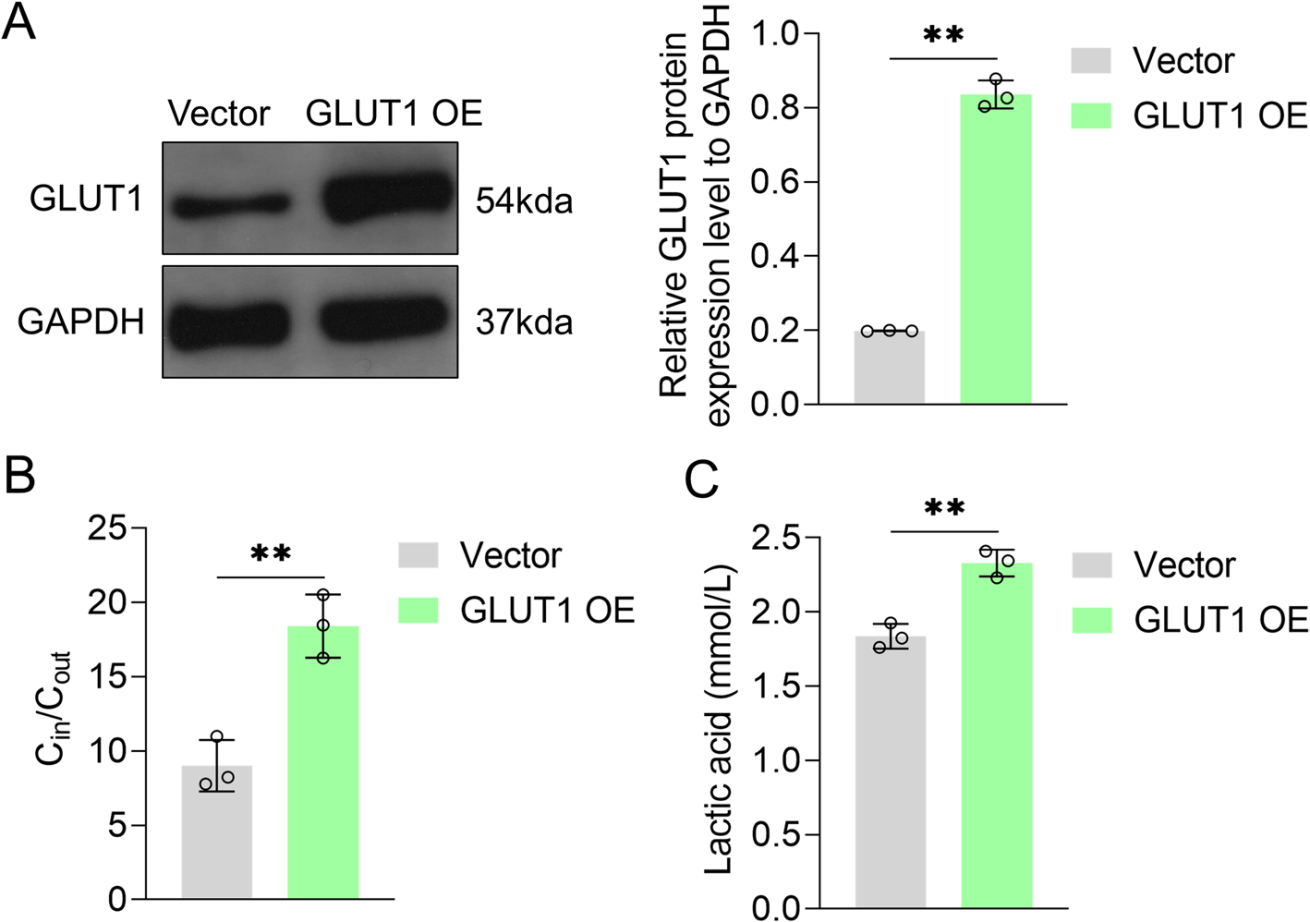
Effects of GLUT1 overexpression plasmid on glycolysis of VFL epithelial cells. **A** Effects of transfection with GLUT1 overexpression plasmid on GLUT1 protein content in VFL epithelial cells, as detected by Western blotting. GAPDH was served as the internal reference. On the right, strip gray values are shown. **B** Effects of transfection with GLUT1 overexpression plasmid on glucose uptake and **C** lactic acid content. *t* test was used for statistical analysis. **P < 0.01

After transfection with GLUT1 overexpression plasmid, GLUT1 protein expression levels were found to be significantly upregulated in VFL epithelial cells (*P* < 0.01, Fig. 5A). Furthermore, in comparison with transfection with control plasmid, that with GLUT1 overexpression plasmid enhanced ^18^F-FDG uptake and lactate secretion by VFL epithelial cells (*P* < 0.01, Fig. 5B, C), indicating that GLUT1 enhanced glycolysis.

**Fig. 6:**
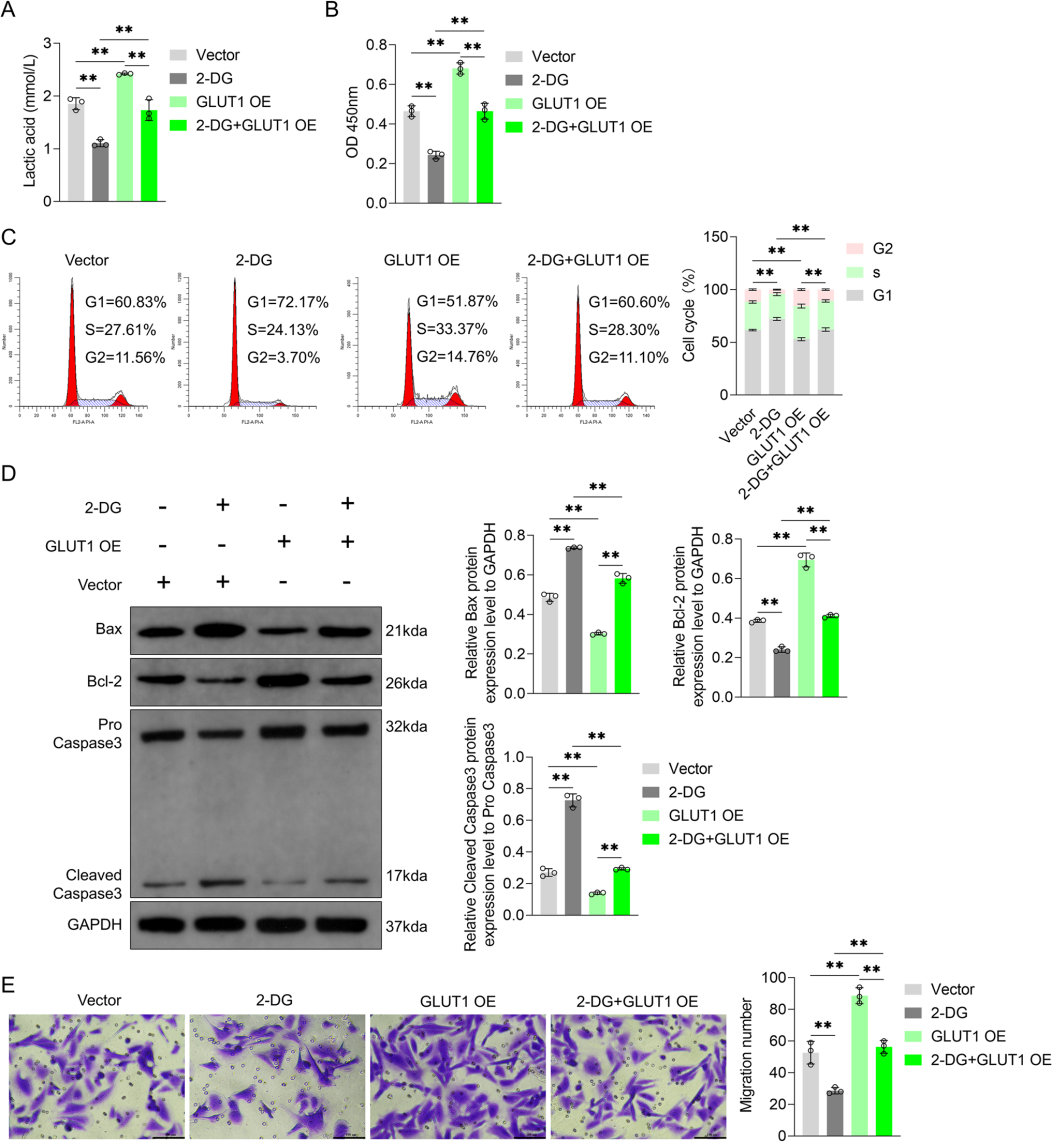
Effects of GLUT1 overexpression plasmid and 2-deoxy-glucose (2-DG) on glycolysis and growth and migration of VFL epithelial cells. **A** Effects of GLUT1 overexpression plasmid and 2-DG (5 mM) on the content of lactic acid and **B** activity of VFL epithelial cells. **C** PI dye combined with flow cytometry was used to detect the effects of GLUT1 overexpression plasmid and 2-DG (5 mM) on cell cycle distribution. **D** Effects of GLUT1 overexpression plasmid and 2-DG (5 mM) on caspase-3 shear and Bax and Bcl-2 expression were detected by Western blotting. GAPDH was served as the internal reference. On the right, strip gray values are shown. **E** Transwell chamber assay was performed to detect the effects of GLUT1 overexpression plasmid and 2-DG (5 mM) on the migration ability of VFL epithelial cells. Scale, 100 μm. One-way ANOVA was used for statistical analysis. **P < 0.01

Glycolysis inhibitor 2-deoxy-glucose (2-DG) significantly reduced lactic acid content of VFL epithelial cells (*P* < 0.01, Fig. 6A). Besides, lactate secretion by the GLUT1-overexpressed cells combined with 2-DG treatment was significantly lower than that by those transfected with GLUT1 overexpression plasmid (*P* < 0.01, Fig. 6A), indicating that 2-DG blocked GLUT1-mediated glycolysis.

In comparison with transfection with control plasmid, the activity of 2-DG-treated VFL epithelial cells showed a significant decrease (*P* < 0.01, Fig. 6B). Moreover, compared to transfection with control plasmid, that with GLUT1 overexpression plasmid significantly increased the activity of the cells (*P* < 0.01), but this effect was reversed by 2-DG (*P* < 0.01, Fig. 6B). The findings of flow cytometry analyses revealed that 2-DG reduced the proportion of the cells in the S phase and increased that of those in the G1 phase in the VFL epithelial cells transfected with control plasmid (*P* < 0.01, Fig. 6C); moreover, 2-DG blocked the promoting effect of the transfection with GLUT1 overexpression plasmid on the proportion of the cells in the S phase and blocked the reduction effect of the transfection with GLUT1 overexpression plasmid on the proportion of the cells in the G1 phase (*P* < 0.01, Fig. 6C). Western blotting results showed that 2-DG promoted caspase-3 cleavage and Bax expression but reduced Bcl-2 protein content in VFL epithelial cells (*P* < 0.01, Fig. 6D). Besides, transfecting VFL epithelial cells with GLUT1 overexpression plasmid significantly inhibited the content of cleaved caspase-3 and Bax and increased the expression of Bcl-2 (*P* < 0.01, Fig. 6D), but this effect was partially reversed by 2-DG (*P* < 0.01, Fig. 6D). The results of transwell chamber and CCK-8 assays were similar. 2-DG significantly inhibited the migration ability of VFL epithelial cells transfected with control plasmid (group 2 vs. 1, *P* < 0.01, Fig. 6E). GLUT1 overexpression plasmid, on the other hand, enhanced the migration ability of these cells (*P* < 0.01), but 2-DG partially blocked the regulatory effect (*P* < 0.01, Fig. 6E). Collectively, these data indicated that GLUT1 promoted glycolysis to consequently enhance the growth and migration capabilities of VFL epithelial cells.

### Acidified pepsin promoted Hexokinase-II (HK-II) expression and mitochondrial translocation in VFL epithelial cells

**Fig. 7:**
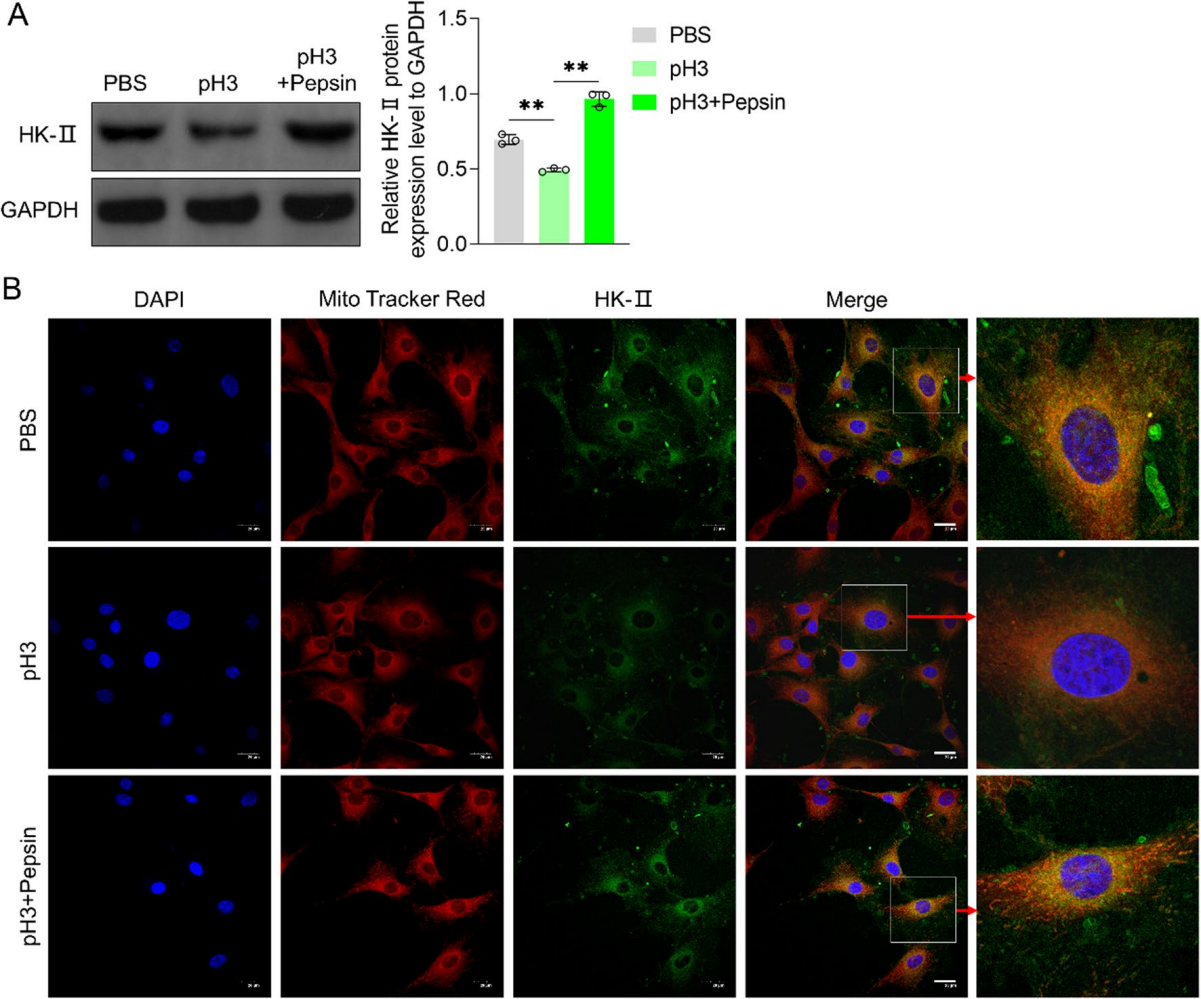
Effects of acidified pepsin on Hexokinase-II (HK-II) expression and mitochondrial translocation in VFL epithelial cells. **A** Effects of acidified medium (pH 3) and acidified pepsin (pH 3, 0.1 mg/mL) on the amount of HK-II protein, as detected by Western blotting. GAPDH was served as the internal reference. On the right, strip gray values have been plotted. **B** Effects of acidified medium (pH 3) and acidified pepsin (pH 3, 0.1 mg/mL) on fluorescence expression intensity of HK-II in the mitochondria of VFL epithelial cells, as observed under a laser confocal microscope. Scale, 20 μm. One-way ANOVA was used for statistical analysis. **P < 0.01

Western blotting results indicated that in acidified medium, HK-II expression was significantly lower than that in control medium (*P* < 0.01, Fig. 7A). Furthermore, HK-II protein expression in acidified pepsin-treated VFL epithelial cells was significantly higher than that in acidified medium (*P* < 0.01, Fig. 7A). In comparison with acidified medium, the fluorescence expression intensity of HK-II was higher in acidified pepsin-treated VFL epithelial cells (Fig. 7B), which was consistent with Western blotting data. Compared to control medium, the fluorescence expression intensity of HK-II in Mito-Tracker Red^+^ aggregates of VFL epithelial cells in acidified medium was lower. Moreover, compared to acidified medium, the fluorescence expression intensity of HK-II in Mito-Tracker Red^+^ aggregates of acidified pepsin-treated VFL epithelial cells was higher (Fig. 7B). These results showed that acidified pepsin enhanced HK-II content in the mitochondria of VFL epithelial cells.

## Discussion

LPR is reportedly associated with laryngeal cancer [32–34]. Pepsin, a key non-acid component of LPR [35, 36], is the first protease that food proteins come across in the digestive tract. Pepsin has 330–350 amino acid residues, mainly with β-sheets. In our previous study, we reported that in patients with VFL, pepsin expression significantly increased with an increase in the grade of dysplasia [8]. Pepsin evidently enters the laryngeal mucosa with LPR. Ao et al*.* reported that long-term administration of pepsin to larynx led to hyperplasia of mice laryngeal epithelium [11]. In this study, pepsin content increased in VFL with the increase in the grade of dysplasia. In addition to that, pepsin was also moderately expressed in control tissues. The reason was as below: control tissues were collected from adjacent tissues of VFL patients and the adjacent tissues of VFL were also exposed in the complex microenvironment which was different from normal tissues. Previous study found that acidified pepsin promoted the growth of hypopharyngeal cancer cells (FaDu cells), presumably by inducing G1/S transition [37]. Pepsin has also been found to enhance the migration ability of laryngeal cancer cells [9]. Our results validated that in case of VFL, pepsin protein content increased with an increase in the grade of dysplasia. Furthermore, we found that acidified pepsin promoted the growth of VFL epithelial cells, which might be related to G1/S transition. Acidified pepsin also seemed to play an antiapoptotic role in VFL epithelial cells by increasing mitochondrial membrane potential, inhibiting caspase-3 shear and Bax expression, and upregulating Bcl-2 expression. Overall, we found that acidified pepsin enhanced the growth and migration ability of VFL epithelial cells. Our results suggested that pepsin induced malignant transformation of VFL epithelial cells by promoting their growth and migration.

Laryngeal cancer tissues and cells always appear increased glycolysis, and the inhibition of glycolysis has been found to enhance radiosensitivity and suppress cancer progression [13, 38, 39]. Herein, we observed that acidified pepsin-treated VFL epithelial cells showed significantly increased glucose uptake, lactate secretion, and ECAR values. 2-DG treatment reduced the growth and migration of these cells. GLUT1, a glucose transporter, plays a key regulatory role in glycolysis. The inhibition of GLUT1 expression has been reported to block glycolysis and improve radiosensitivity, and also inhibit cell proliferation and glucose uptake in case of laryngeal cancer [19–21, 40, 41]. Acidified pepsin can increase GLUT1 protein expression in VFL epithelial cells [7]; our findings further validated this observation. GLUT1 upregulation in VFL epithelial cells promoted glucose uptake, lactate secretion, cell activity, S phase ratio, and migration capability, which could be partially blocked by 2-DG. It appears that acidified pepsin enhances glycolysis by upregulating GLUT1 expression. Further studies are warranted to comprehensively investigate the blocking effect of inhibition of acidified pepsin-mediated GLUT1 expression on glycolysis as well as growth and migration of VFL epithelial cells.

The enhancement of glycolysis is accompanied by an increase in lactic acid levels, leading to an increase in intracellular [H]^+^ levels. The inhibition of extracellular transport of [H]^+^ can lead to acidification of cancer cells and promote their apoptosis [42]. Monocarboxylate transporters (MCTs) transfer monocarboxylic acids, such as ketones, lactic acid, pyruvate, and short chain fatty acids. MCT4, in particular, transports short chain sugars, such as pyruvate and lactic acid, so as to maintain intracellular pH levels and glycolysis; inhibition of its activity can lead to the intracellular accumulation of lactic acid and [H]^+^, consequently resulting in glycolysis inhibition and cancer cell death [43]. Knockout of MCT4 attenuates 4-nitroquinoline-1-oxide-induced oral cancer formation [44]. A study found MCT4 expression in laryngeal cancer tissue to be significantly higher than that in normal mucosal tissue [45]. In this study, we found that acidified pepsin significantly upregulated MCT4 protein expression in VFL epithelial cells. We believe that the increased expression of MCT4 is related to acidified pepsin-mediated increase in glycolysis and growth and migration of VFL epithelial cells.

HK-II is the first-rate limiting enzyme in glycolysis. It has been reported that inhibiting HK-II expression blocks glycolysis and improves the radiosensitivity of laryngeal cancer cells [16]. Furthermore, Grimm et al*.* has reported that HK-II expression in normal oral mucosa, mucosal atypical hyperplasia, squamous intraepithelial neoplasia stage I–III, and invasive squamous cell carcinoma cells gradually increase with the aggravation of lesions [46]. Herein, we found that acidified pepsin increased HK-II expression in VFL epithelial cells, implying that acidified pepsin-mediated glycolysis was related to the expression of HK-II. Voltage-dependent anion channels (VDACs) exist on the outer membrane of mitochondria, which participate in the exchange of ions and metabolites between the cell membrane and mitochondria [47, 48]. Mitochondrial permeability transition pore opening, which is associated with mitochondrial-mediated apoptosis, is considered to be triggered by VDAC-mediated Ca^2+^ entry [47, 49–51]. VDAC1 plays a vital regulatory role in cell survival and death [52]. HK-II reportedly combines with VDAC, which inhibits VDAC activity and mitochondrial permeability transition pore opening, and consequently, apoptosis is prevented [53]. In this study, mitochondrial HK-II was increased in acidified pepsin-exposed VFL epithelial cells; we believe that acidified pepsin blocks VDAC activity by increasing HK-II content in mitochondria, participating in the regulation of VFL epithelial cell apoptosis.

The enhancement of glycolysis in cancer cells is often accompanied by a decrease in oxidative phosphorylation capacity [54]. Focal adhesion kinase not only promotes glycolysis in tumor cells, but also inhibits mitochondrial respiration [55]. Kaposi sarcoma-associated herpesvirus has been reported to promote cell malignant transformation accompanied by reduced oxidative phosphorylation [56]. We found that acidified pepsin reduced oxidative phosphorylation of VFL epithelial cells. A reduction in the activity of mitochondrial respiratory chain complex I evidently increases NADH levels, decreases NAD^+^/NADH ratio, and promotes pyruvate conversion to lactate; moreover, decreased NAD^+^/NADH ratio facilitates pyruvate into the glycolytic pathway [57]. We herein found that acidified pepsin reduced the activity of mitochondrial respiratory chain complex I in VFL epithelial cells. It seems that acidified pepsin-mediated enhanced glycolysis of VFL epithelial cells is associated with the decrease in mitochondrial respiratory chain complex activity and oxidative phosphorylation. It has been reported that mitochondrial damage can lead to the impairment of the mitochondrial function, resulting in the weakening of mitochondrial respiration and oxidative phosphorylation [58]. Pepsin can reportedly cause mitochondrial dysfunction in epithelial cells, including hypopharyngeal epithelial cells [59, 60]. Therefore, we believe that pepsin mediates decreased oxidative phosphorylation of VFL epithelial cells, which may be related to mitochondrial damage.

## Conclusions

Acidified pepsin promotes the transition from oxidative phosphorylation to glycolysis by reducing the activity of mitochondrial respiratory chain complex I and enhancing the expression of HK-II and GLUT1, which eventually increases the growth and migration of VFL epithelial cells. In addition, the antiapoptotic regulation of acidified pepsin on VFL epithelial cells may be related to HK-II expression in mitochondria.

## Supplementary Information

Below is the link to the electronic supplementary material.

Supplementary file1 (DOCX 208 KB)

## Data Availability

The authors confirm that the data supporting the findings of this study are available within the article [and/or] its supplementary materials.
