# Supplementary material for: Pepsin enhances glycolysis to promote malignant transformation of vocal fold leukoplakia epithelial cells with dysplasia
Source: Eur Arch Otorhinolaryngol. 2022 Nov 16;280(4):1841–54. doi: 10.1007/s00405-022-07729-5 (PMC9988773; doi:10.1007/s00405-022-07729-5)

**Figure S1. Effects of pepsin in acidified medium and pepsin in normal medium on cell viability of VFL epithelial cells.** Effects of acidified pepsin (pH 3 or 5) at different concentrations (pH 3 or 5; 0, 0.01, 0.05, 0.1 and 0.5 mg/mL), pepsin in normal medium (pH 7) at different concentrations (pH 7; 0, 0.01, 0.05, 0.1 and 0.5 mg/mL), and inactivated pepsin in normal medium (pH 7) at different concentrations (pH 7; 0, 0.01, 0.05, 0.1 and 0.5 mg/mL) on the cell viability of VFL epithelial cells were detected by CCK-8 assay. **, ^^, ##P < 0.01.


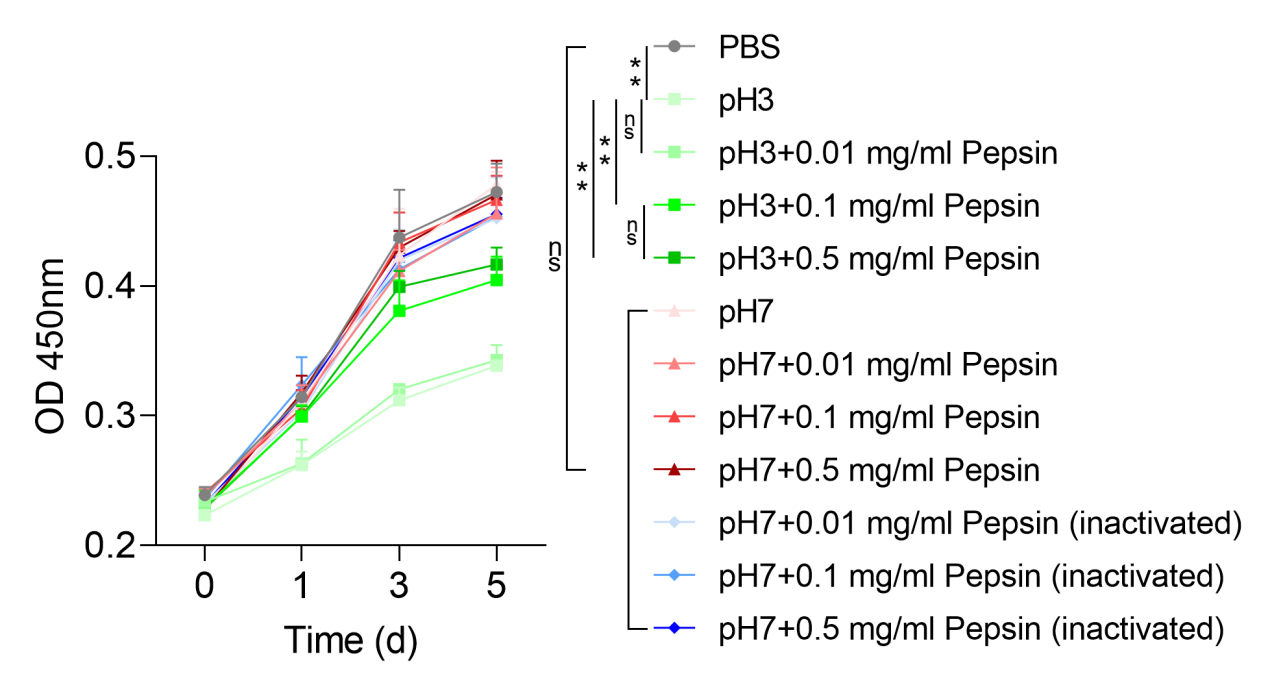

Supplement: Supplementary file 1 — Supplementary file1 (DOCX 208 KB) [file 405_2022_7729_MOESM1_ESM.docx]
